# The effect of Macro and Nano‐emulsions of cinnamon essential oil on the properties of edible active films

**DOI:** 10.1002/fsn3.1946

**Published:** 2020-10-25

**Authors:** Reza Fattahi, Babak Ghanbarzadeh, Jalal Dehghannya, Mohammadyar Hosseini, Pasquale M. Falcone

**Affiliations:** ^1^ Department of Food Science and Technology Faculty of Agriculture University of Tabriz Tabriz Iran; ^2^ Department of Food Engineering Faculty of Engineering Near East University Mersin Turkey; ^3^ Department of Food Science and Technology Faculty of Agriculture University of Ilam; ^4^ Department of Agricultural, Food and Environmental Sciences University Polytechnical of Marche Ancona Italy

**Keywords:** cinnamon essential oil, emulsified film, Macro‐emulsion, Nano‐emulsion

## Abstract

The effect of Nano‐emulsion (NE) and Macro‐emulsion (ME) of cinnamon essential oil (CEO) on the properties of carboxymethyl cellulose (CMC)‐based films was investigated. MEs (diameters of 242–362 nm) and NEs (diameters of 59–80 nm) of CEO were produced through Ultra‐Turrax and Ultrasonication, respectively. The scanning electron microscopy (*SEM*) and atomic force microscopy (AFM) images showed different morphologies in the films containing ME and NE, also a denser and more uniform microstructure was observed in the NE films in comparison with the ME ones. The higher stability of NE in the CMC matrix, increased the thickness of the resulted films. The water vapor permeability (WVP) was increased from 2.59 × 10^–9^ g/ms Pa in the control film to 4.43 × 10^–9^ g/m s Pa in the ME film, and decreased to 1.80 × 10^–9^ g/ms Pa in the NE film. Adding CEO led to more flexible films with enhanced strain at break (SAB) from 53.56% in the control film to 80% and 94.77% in the ME and NE films, respectively. The antifungal indices against *A. niger* and *M. racemous* were 14.16% and 20.82% in the ME films, and were improved to 18.81% and 25% in the NE ones.

## Graphical abstract

The effects of Nano‐emulsion (NE) and Macro‐emulsion (ME) of cinnamon essential oil (CEO) on the properties of carboxymethyl cellulose (CMC) based films were investigated. Adding CEO led to more flexible films with enhanced strain at break (SAB) from 53.56% in the control film to 80% and 94.77% in the ME and NE films, respectively. The antifungal index against *A. niger* and *M. racemous* were 14.16% and 20.82% in the ME films and were improved to 18.81% and 25% in the NE ones.

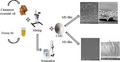

## 1 INTRODUCTION

Over the last few years, the tendency to use biodegradable polymers in food packaging has grown due to increased awareness regarding ecological and environmental problems and contamination of natural resources caused by nondegradable petrochemical‐based polymers (Al‐Tayyar et al., 2020; da Silva Filipini et al., 2020). Considering this, two types of biodegradable polymers have been investigated: edible and nonedible biopolymers. Carbohydrates and proteins or their combination with lipids are usually used for producing edible biopolymers (Noshirvani, Ghanbarzadeh, Gardrat, et al., 2017; Noshirvani, Ghanbarzadeh, Rezaei Mokarram, et al., 2017).

Carboxymethyl cellulose (CMC) is a biodegradable semi‐synthetic biopolymer. It is a linear negative‐charged polysaccharide due to having many hydrophilic carboxyl groups (Oun & Rhim, 2020). CMC produces high viscosity solution and transparent films with desirable properties from consumer point of view. The CMC‐based films show good barrier properties against gases and lipophilic compounds, while showing poor inhibition against water vapor permeation, similar to other polysaccharide and protein‐based films (Ghanbarzadeh & Almasi, 2011; You et al., 2016). In order to overcome these disadvantages and improve packaging properties, a number of approaches have been studied such as combination with other compatible biopolymers, using hydrophobic compounds (such as lipid and essential oil), cross‐linking agents and Nano‐fillers (Noshirvani, Ghanbarzadeh, Gardrat, et al., 2017; Noshirvani, Ghanbarzadeh, Rezaei Mokarram, et al., 2017; Sahraee et al., 2017a).

Mostly, synthetic antimicrobial and antioxidant compounds are directly added into the food products in order to inhibit microbial growth. This method may lead to the inactivation or evaporation of active agents and their rapid migration into the food. Hence, the antimicrobial activity may be quickly lost by dilution below active concentration, and also the flavor of the food may change (Seibert et al., 2019). The incorporation of an antimicrobial compound into a polymer matrix and the production of active packaging has the potential to cause the gradual release of active agents, which may be a promising approach to inhibit the microbial growth (Noshirvani et al., 2018; Sahraee et al., 2017b).

In order to meet consumers' demands for more natural materials and extend product shelf life, research has been focused on the incorporation of natural shelf life extenders such as plant extracts, essential oils (EOs) and bacteriocins into the bio‐based packaging (Acevedo‐Fani et al., 2015; Bagde & Nadanathangam, 2019). EOs are classified as generally recognized as safe (GRAS) (Dashipour et al., 2015). However, direct EO incorporation into foods is restricted by undesirable sensory changes, low water solubility, volatility, and interaction with other food components (Hashemi Gahruie et al., 2017).

Cinnamon essential oil (CEO) is a mixture of aldehyde, phenol and terpene active compounds, exhibiting antioxidant characteristics and a wide spectrum of antimicrobial properties against fungal spoilage (Sun et al., 2020). CEO has been approved by the World Health Organization (WHO) as a nontoxic additive and flavoring agent, therefore CEO is widely applied in many food formulations, meeting consumers’ demand for a desirable flavor (Chen et al., 2016; Noshirvani, Ghanbarzadeh, Gardrat, et al., 2017; Noshirvani, Ghanbarzadeh, Rezaei Mokarram, et al., 2017; Ojagh et al., 2010; Otoni, Moura, et al., 2014; Otoni, Pontes, et al., 2014).

The droplet size distribution in Nano‐emulsions (NE) is under 100 nm (Shadman et al., 2017; Zhang et al., 2017). Using essential oils as a Nano‐emulsion in biopolymer films has shown important characteristics in comparison with Macro‐emulsions, such as increased kinetic stability, protection from degradation and evaporation, improved physical and mechanical properties of the film and enhanced biological accessibility due to increasing the surface area which in turn facilitates migration of Nano‐sized droplets via cell membrane of microorganisms (Acevedo‐Fani et al., 2015; Mendes et al., 2020; Otoni et al., 2016; Otoni, Moura, et al., 2014; Otoni, Pontes, et al., 2014; Zhang et al., 2017).

Regarding literature review, only few studies have been focused on Nano‐emulsion incorporated films produced from essential oil and biopolymer: chitosan films and cinnamaldehyde Nano‐emulsions (Chen et al., 2016), pectin or papaya puree edible films and cinnamaldehyde Nano‐emulsions (Otoni et al., 2016), fish gelatin films and cinnamon essential oil Nanoliposomes (Wu et al., 2015), basil seed gum films and *Zataria multiflora* essential oil Nano‐emulsions (Hashemi Gahruie et al., 2017), alginate films and lemongrass essential oil (Riquelme et al., 2017), pectin films and marjoram essential oil Nano‐emulsions (Almasi et al., 2020), corn starch films and *Zataria multiflora* essential oil Nano‐emulsions (Amiri et al., 2019), sodium caseinate films and cinnamon essential oil Nano‐emulsions (Ranjbaryan et al., 2019).

EOs encapsulated in smaller droplets have shown improved antifungal activities against *Fusarium graminearum* in cereals (Wan et al., 2019). Lee et al. (2019) studied the addition of encapsulated oregano essential oil into HPMC‐based films produced by casting and found that emulsion droplet size significantly affected the physical properties and antibacterial activity of the films (Lee et al., 2019). Hazelnut meal protein and clove EO Nano‐emulsion active films were reported to have improved in vitro antibacterial properties against *E. coli*, *Staphylococcus aureus*, *Listeria monocytogenes*, *Bacillus subtilis*, and *Pseudomonas aeruginosa* when droplets were smaller (Gul et al., 2018). In this study, a disk diffusion test indicated that inhibition zone values were increased significantly by decreasing EO droplet size from 248 to 160 nm.

To our knowledge, there is no study on comparing the effect of Nano and Macro‐emulsions of cinnamon essential oil (CEO) on the physicochemical and antifungal properties of the active biopolymer‐based films. Therefore, the objective of this research was (a) producing emulsified CMC‐based films from CEO Macro‐emulsions (ME) and Nano‐emulsions (NE) prepared by using Ultra‐Turrax and Ultrasonication (b) to compare the effects of these two types of emulsified films on some physical features of the resulted films such as mechanical, microstructural and barrier‐related properties (c) and to study the in vitro efficiency of these films against *Aspergillus niger* and *mucor racemous*.

## 2 MATERIALS AND METHODS

### 2.1 Materials

Carboxymethyl cellulose (CMC), with an average molecular weight of 41,000 g/mol (practical grade) was obtained from Caragum Parsian. Analytical grade glycerol was purchased from Dr. Mojallali Chemical Laboratories. Polysorbate 80 (Tween 80) was obtained from Merck and used as a surfactant. Potato dextrose agar (PDA) was purchased from QUELAB. Cinnamon essential oil (*Cinnamomum zeylanicum*) was supplied by Exire Gole Sorkh Pharmaceutical Co. *Aspergillus niger* and *Mucor racemous* were obtained from the Persian Type Culture collection. Double‐distilled water was used in all emulsions and film‐forming solutions.

### 2.2 Preparation of cinnamon essential oil emulsions

#### 2.2.1 Macro (ME) and Nano (NE)‐emulsions

Macro‐emulsions (ME) were made by adding different amounts (0.125, 0.25 or 0.5 g) of Tween 80, equal with 50% w/w surfactant oil ratio (SOR), to 20 ml of double‐distilled water, followed by homogenization by Ultra‐Turrax (JANKE & KUNKEL), at 12,298 *g* for 1 min. Then, cinnamon essential oil (CEO) was added (0.2427, 0.4854 or 0.9708 ml), equal with 0.2427%, 0.4854% and 0.9708% v/v (CEO/ film‐forming emulsion), to each solution and mixed again for 2 min. The prepared ME were sonicated at 20 KHz frequency, 400 W input power and 70S amplitude for 10 min by Ultrasonicator (FAPAN) to obtain Nano‐emulsions. In order to prevent the increase of emulsion temperature during sonication, the solutions were kept in an ice‐water bath.

#### 2.2.2 Droplet size and distribution in CEO emulsions

The average droplet size and PdI of both Macro (ME) and Nano (NE)‐emulsions of cinnamon essential oil (CEO) were calculated by dynamic light scattering (DLS) in a Zetasizer, Nano ZS laser diffractometer (Malvern instruments). The emulsions were first diluted in ultrapure water to (1:10) of their initial concentration to avoid multiple scattering effect and interdroplet interactions (Otoni, Moura, et al., 2014; Otoni, Pontes, et al., 2014). A polydispersity index was described as the size distribution of droplets, according to which values close to 0 indicate a homogenous dispersion. The cumulant mean (*Z*‐average) size was defined as the scattering intensity weight. The Z‐average was calculated using the following equation Equation 1:

(1) $$D_{z}\approx \frac{\sum S_{i}}{\sum \left(\frac{S_{i}}{D_{i}}\right)}$$

where, *D*_Z_ is mean diameter, *D*_i_ is diameter of each droplet and *S*_i_ is intensity ($S_{i}\approx D_{i}^{6}$).

#### 2.2.3 ζ ‐potential of film‐forming solutions

The zeta potential of film‐forming solutions loaded with ME or NE was calculated by dynamic light scattering (DLS) in a Zetasizer (Malvern instruments). The ζ‐potential is the electrical charge of the slipping plan at the interface of CEO droplets dispersed in the biopolymer solution.

### 2.3 Formation and characterization of emulsified films

#### 2.3.1 Preparation of emulsified films

Films were prepared as described by Dashipour et al. (2015) with slight modifications. First, 80 ml of double‐distilled water was heated in a water bath until temperature increased to 85°C. Then, 1.5 g of CMC powder was added to hot water and mixed by magnetic stirrer (800 rpm) at 85°C for 60 min. After that, glycerol was added at 0.75 w/w of CMC as a plasticizer and mixing continued for 10 min. Then, the solution temperature was reduced to 60°C in order to prevent the destruction of CEO active compounds. Having done that, the solution was mixed with 20 ml of Macro and Nano‐emulsion solutions prepared in the previous section and agitated with magnetic stirrer (500 rpm) at 60°C for 30 min to obtain homogeneous solutions. The film‐forming solutions were degassed with vacuum pump (DV‐3E 250, JB), at ambient condition for 5 min. Then, 100 ml of the emulsified film‐forming solutions were poured into flat‐surfaced Teflon plates (PTFE) at the dimension of 15 × 15 cm and dried in an oven at 40°C for 18 hr to cast the films.

#### 2.3.2 Scanning electron microscopy (*SEM*)

Microstructural analysis of the surface and the cross‐section of emulsified films incorporated with different concentrations of CEO Macro and Nano‐emulsions were carried out by Scanning Electron Microscopy (FEG‐MIRA3, Tescan). The films were fractured under liquid nitrogen and mounted on the specimen holder with copper stubs and then sputtered with gold (DSR1, Nanostructural Coating Co). After coating with a thin layer of gold, the films were imaged at an accelerating voltage of 10 KV at the 5,000 magnification.

#### 2.3.3 Atomic force microscopy (AFM)

The surface topography of emulsified CMC‐based films was observed by Atomic Force Microscopy (Nanosurf). A sharpened cantilever was placed on top of the films, and 8 μm × 8 μm images were scanned and then the images were analyzed by Nanosurface Mobile S software (Version 2.2.2.1) to determine the Sa (average of the absolute value of the height deviations from a mean surface) and Sq (root‐mean square average of height deviations taken from the mean data plane) as statistical parameters of the surface roughness index. The measurements were carried out on the top casting surface of the films.

#### 2.3.4 Film thickness

The thickness of emulsified films was determined with a hand‐held digital micrometer (Guanglu), with a precision of 0.01 mm. The values were taken at ten random positions for each film. The average values were used to determine the water vapor permeability (WVP) and mechanical properties.

#### 2.3.5 Mechanical properties

Ultimate tensile strength (UTS) and strain at break point (SAB) were determined using a universal testing machine (Sanaf). Dried films were cut into dumbbell shape and testing was performed according to the guidelines of American Society for Testing and Material method D882‐91 (ASTM, 2012). Films were cut in 8 × 0.50 cm dumbbells preconditioned in three replicates for each film at ambient temperature and 53% RH in the desiccators containing magnesium nitrate‐saturated solutions for 24 hr before measurements. The films were mounted parallel with an initial grip machine with the length of 5 cm and the cross‐head speed was set at 0.50 cm/min. UTS and SAB were evaluated using the following equations Equations 2 and 3:

(2) $$\text{UTS}=\frac{F\max}{A}$$

(3) $$\text{SAB}=\left(\frac{\Delta L}{L_{0}}\right)\times 100$$

where, *F*max is the maximum force needed to separate the biopolymer into pieces (N), *A* is the cross‐section (thickness × width) of the films (m^2^), *L*_0_ is the primary gage separation (0.50 cm) and Δ*L* is the length difference between the initial and the stretched length of the film in break point.

#### 2.3.6 Water vapor permeability (WVP)

The WVP of the films was calculated by a gravimeter following ASTM method (E96‐95) with slight modifications (ASTM, 1995). Circular test cups with a mean internal diameter of 1.50 cm, containing 3 g of anhydrous calcium sulfate (0% RH inside a cup), were sealed by the test films with a diameter slightly larger than the internal diameter of the cup (0.017 cm^2^ circular disk area). Each cup was placed into a desiccator containing sodium chloride (75% RH inside a desiccator) solution (Merck) at 25°C. Under this condition, the driving force was 1.75355 KPa, expressed as water vapor partial pressure. To ensure that the solution remained saturated during the experiment, a small sediment of sodium chloride was left on the bottom of the desiccator. Then, the weights of the cups were recorded by a sensitive digital balance with the accuracy of 0.0001 g, every 24 hr until the steady state was reached. The water vapor transmission rate (WVTR), defined as the slope of the steady‐state period of the curve of the weight gain as a function of the time (g/s), was determined by the linear regression divided by the transfer area (m^2^). WVP (g/ms Pa) was calculated using the following equation Equation 4:

(4) $$\text{WVP}=\frac{\left(\text{WVTR}\times \Delta x\right)}{\Delta P}$$

where, Δ*x* is the average film thickness (m), and Δ*P* is the partial vapor pressure difference of the atmosphere with CaSO_4_ and NaCl (1.75355 kPa). All measurements were done in three replicates.

#### 2.3.7 Antifungal properties

Antifungal activity of the films was determined using disk diffusion method as described by Noshirvani, Ghanbarzadeh, Gardrat, et al. (2017), Noshirvani, Ghanbarzadeh, Rezaei Mokarram, et al. (2017) with slight modifications. *A. niger* (ATCC 64974) and *M. racemous* (IBRC‐M number 30117), initial suspensions were activated by inoculating on Potato Dextrose Agar (PDA) at 25°C. After observing spores, they were diluted by the sterile swabs in double‐distilled water. Then, 0.1% of Tween 80 was added to reduce surface tension and facilitate dispersion in distilled water. After that, the spores were counted by a Neubarer cell under the microscope until reaching the concentration of 10^6^ CFU/ml for fungi. Having done that, PDA solution was poured into plates with 80 mm diameter. After the solidification of the media, the diluted spore solutions were spread on the media surface. The films were then cut into a disk shape of 8 mm diameter by using a sterile puncher and placed on the surface media which had been previously smeared with 100 μl of inoculums containing approximately 10^6^ CFU/ml of the tested molds. The plates were incubated at 25°C for 24 hr and then the diameter of the inhibition zones around the film disks on solid media was measured with a digital micrometer. After that, the Antifungal Index (A.I %) was calculated using the diameter inhibition of the films for Macro and Nano‐emulsions using the equation below Equation 5:

(5) $$A\text{.I}=\left(\frac{D_{i}}{D_{p}}\right)\times 100$$

where, *D*_i_ is the diameter of inhibition zone and *D*_p_ is the diameter of petri dish (80 mm).

### 2.4 Statistical analysis

Data were analyzed with SPSS (Version 16.0 for Windows) software by one‐way analysis of variance (ANOVA). Duncan's multiple range test was used for mean comparison among the data at 5% (*p* < .05). Data were expressed as mean ± standard deviation.

## 3 RESULTS AND DISCUSSION

### 3.1 Droplet size and distribution in CEO emulsions

The size distribution of the essential oil droplets incorporated in the emulsified films affects film characteristics such as antimicrobial, mechanical, barrier and microstructural properties. (Acevedo‐Fani et al., 2015; Hashemi Gahruie et al., 2017; Otoni, Moura, et al., 2014; Otoni, Pontes, et al., 2014). The intensity‐weighted size average values (*Z*‐average) and the poly dispersity index (PdI) for both ME and NE are shown in Table 1. The PdI is a dimensionless factor indicating the broadness of droplet size in spread (Lemarchand et al., 2003). The mean size and size distribution of droplets could become smaller by enhanced input energies (Otoni, Moura, et al., 2014; Otoni, Pontes, et al., 2014; Perez‐Gago & Krochta, 2001). The Ultra‐Turrax, as a relatively low‐energy mechanical method, was used to produce ME, and resulted droplet sizes were in the range of 242–362 nm. Ultrasonication treatment was used in order to decrease the mean size to a few tens of Nanometers (*D*_z_ < 100 nm), as a result of which, size range of 59–80 nm was obtained. Sonication had a significant effect on the decrease of both mean droplet size (*Z*‐avarage) and size distribution (PdI) of CEO emulsion (*p* < .05). Sonication created interfacial waves and produced microbubbles, the collapse of which resulted in the disruption of oil droplets, reducing them to a Nano‐sized scale (Kentish et al., 2008). Hashemi Gahruie et al. (2017) showed that an increase in sonication time caused a reduction in the distribution and the droplet size of *Zataria multiflora* essential oil emulsion in basil seed gum film‐making solutions.

**Table 1:** Droplet diameters (*Z*‐average), Polydispersity Index (PdI) and ζ‐potential values of Macro and Nano‐emulsions of CEO at different concentrations

Emulsion types	*Z*‐average (nm)	PdI	ζ‐potential (mV)
ME‐CEO 0.2427%	242.07 ± 1.01^c^	0.39 ± 0.04^c^	−55.0 ± 0.30^a^
ME‐CEO 0.4854%	262.96 ± 1.02^b^	0.63 ± 0.03^b^	−65.6 ± 0.10^c^
ME‐CEO 0.9708%	362.17 ± 1.07^a^	0.72 ± 0.02^a^	−61.4 ± 0.25^b^
NE‐CEO 0.2427%	59.19 ± 1.06^e^	0.39 ± 0.01^c^	−69.7 ± 0.35^e^
NE‐CEO 0.4854%	80.08 ± 1.02^d^	0.24 ± 0.02^d^	−68.4 ± 0.20^d^
NE‐CEO 0.9708%	80.02 ± 1.03^d^	0.25 ± 0.06^d^	−70.0 ± 0.30^e^

Note

Values were given as mean ± standard deviations. Different superscripts in the same column indicate significant differences (*p* < .05).

The increase of cinnamon essential oil (CEO) concentration from 0.2427% to 0.4854%, in both Macro (ME) and Nano‐emulsion (NE), led to enhanced droplet size (*p* < .05), attributable to the flocculation of the droplets due to the deficiency of the surfactant for complete coverage of the interface. Similar results were found by Bonilla et al. (2012) who evaluated the effect of basil and bergamot essential oil concentrations on the droplet size in chitosan solutions.

**Figure 1:**
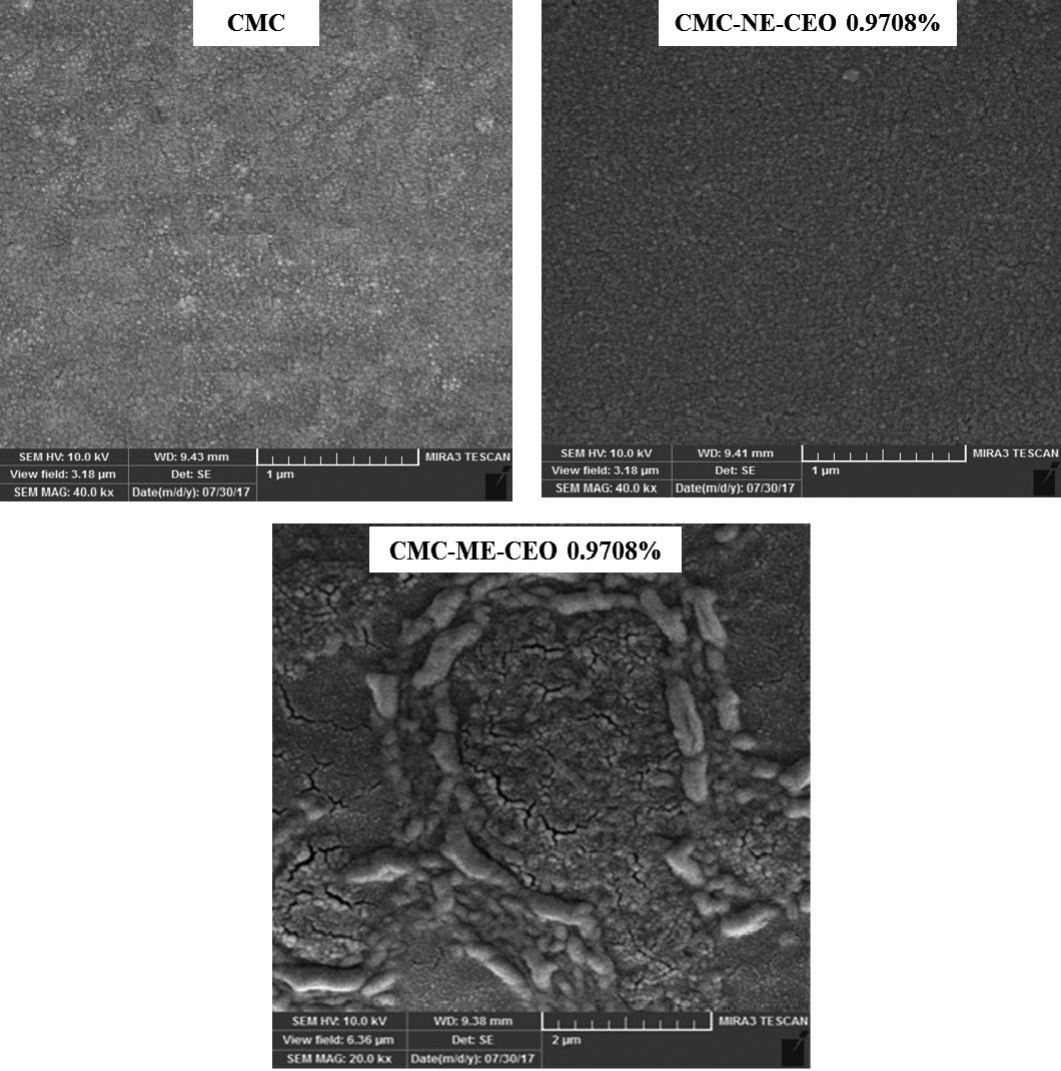
Scanning electron microscopy (*SEM*) images of the surface of the CMC control film and films loaded with ME (CMC‐ME‐CEO) and NE (CMC‐NE‐CEO) of CEO at selected concentration of CEO 0.9708%. CEO, cinnamon essential oil; CMC, carboxymethyl cellulose; ME, Macro‐emulsion; NE, Nano‐emulsion

In addition, increasing the oil concentration from 0.4854% to 0.9708% in the Macro‐emulsion caused a significant increase in the droplet size. Increasing the oil concentration led to a higher viscosity in the dispersion phase. The Ultra‐Turrax homogenizer as a low‐energy method did not have the efficiency to reduce the size of the droplets (Mahdi et al., 2008). As a result, the CEO droplets flocculated. The flocculation was intensified during the film drying due to the increase in the concentration of the CEO in the biopolymer matrix. According to Figure 1, in the film containing ME‐CEO 0.9708%, the flocculation of the oil droplets was visible on the surface of the film. In contrast, increasing the oil concentration from 0.4854% to 0.9708% did not significantly change the droplet size of the Nano‐emulsion. By using Ultrasonic homogenizer as a high‐energy method, the droplet size was not affected by the increase in the dispersion phase viscosity (Lee et al., 2019). On the other hand, the droplet size distribution index did not change significantly when the oil concentration increased from 0.4854% to 0.9708% in the Nano‐emulsion (Table 1). The uniform distribution of droplet size prevents the occurrence of instability phenomena in the Nano‐emulsion such as Ostwald ripening (Sarheed et al., 2020).

### 3.2 ζ‐potential of film‐forming solutions

The ζ‐potential values of the CMC solutions containing Macro and Nano‐emulsions of cinnamon essential oil (CEO) at different concentrations are shown in Table 1. The value of zeta potential in the pure CMC dispersion was −59.2 mV, probably due to the presence of carboxyl functional groups in CMC polymer structure. This value was increased to −61.4 and −70 mV for the samples containing 0.9708% CEO of Macro and Nano‐emulsions, respectively, which could be attributed to the presence of dissociable compounds in the essential oil. The anionic CMC biopolymer could be adsorbed onto the surface of droplets due to the presence of ionizable compounds in the essential oil.

By using higher concentrations of the CEO, negative surface charge increased more in the sample containing NE in comparison with the one containing ME (*p* < .05). This phenomenon may be ascribed to the fact that smaller droplet size in the NE could promote the interactions between the essential oil droplets and the CMC chains, as a result of which a greater amount of CMC could be adsorbed onto the droplet interface (Vargas et al., 2011).

The zeta potential depended on the ionizable components of the essential oil and the size of the droplets (Bonilla et al., 2012; Gul et al., 2018). Increasing the concentration of the CEO increases the ionizable components in the emulsion. In addition, reducing the droplet size improved the CMC‐CEO interactions. Overall, the high concentration of the CEO as well as the small size of the droplets has a synergistic effect on the zeta potential. The Macro‐emulsion containing 0.2427% CEO had the lowest zeta potential. Although the droplet size was small, the amount of the ionizable components was low due to the low concentration of the CEO. However, the Macro‐emulsion containing 0.4854% CEO had a significant increase in zeta potential. Due to the increase in the concentration of the CEO, the amount of ionizable components increased. On the other hand, the average droplet size improved the interactions of CMC‐CEO. But, by increasing the concentration of the CEO to 0.9708%, the zeta potential decreased significantly. Significant increase in droplet size at high CEO concentrations reduced CMC‐CEO interactions.

In the Nano‐emulsion, due to the small size of the droplets (high surface‐to‐volume ratio), the surface of the droplets is completely covered by the CMC. Hence, the effect of the ionizable components of the CEO on the zeta potential was reduced. As a result, the zeta potential did not change with increasing the CEO concentration. But, in the Macro‐emulsion, due to the large size of the droplets (low surface‐to‐volume ratio), the droplet surface was not completely covered by the CMC. As a result, the zeta potential changed significantly with the increase of the ionizable components in the Macro‐emulsion.

Bonilla et al. (2012) reported that positive zeta potential values in a chitosan solution, a cationic biopolymer, were improved by adding essential oil due to an increase in the dissociable compounds. The higher stability of Nano‐emulsions in comparison with Macro‐emulsions in the film‐forming solutions could be due to their relatively higher number of charged groups and hence higher electrostatic repulsion on the CEO droplets surface, preventing flocculation, coalescence and creaming during drying of the films (McClements, 2005).

### 3.3 Scanning electron microscopy (*SEM*)

**Figure 2:**
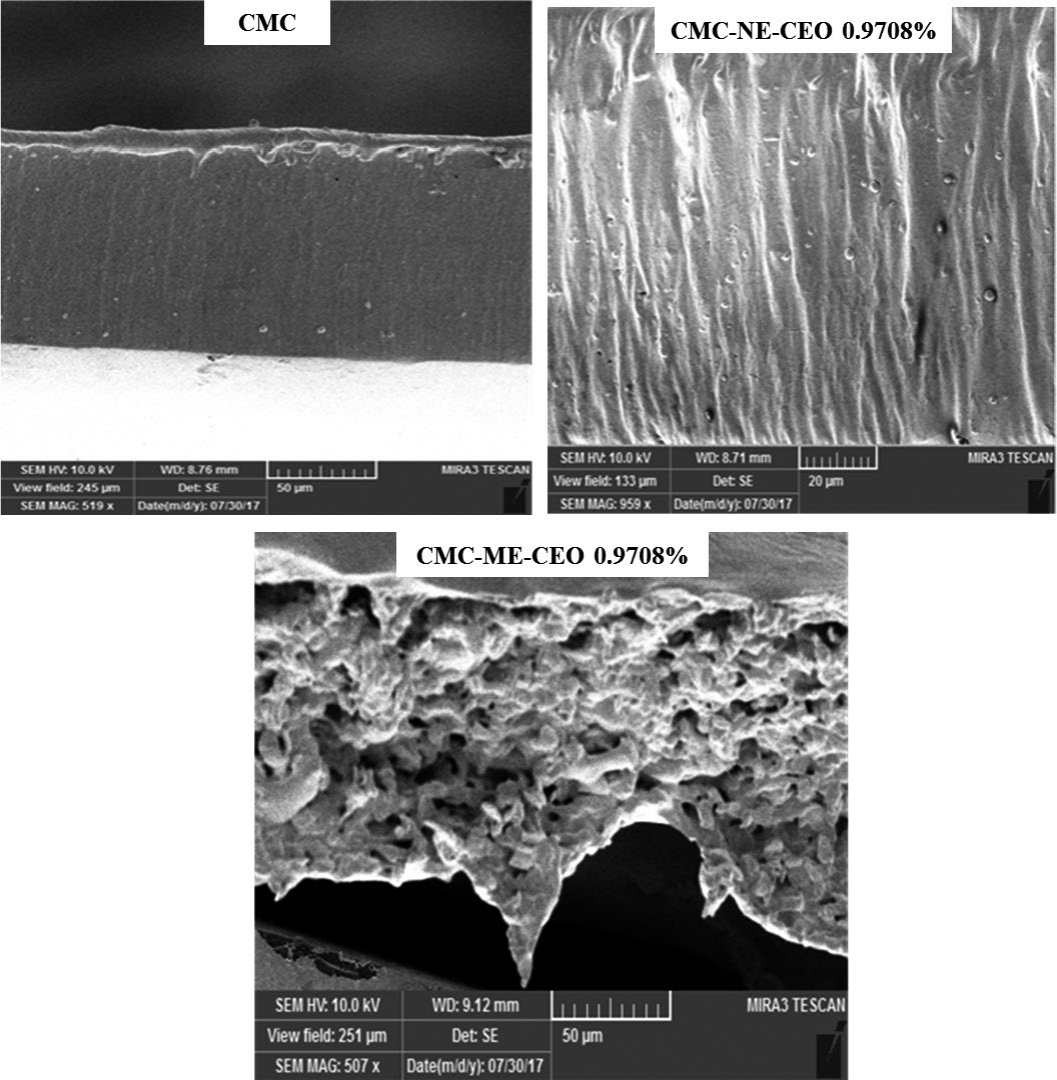
Scanning electron microscopy (*SEM*) images of the cross‐section of the CMC control film and films loaded with ME (CMC‐ME‐CEO) and NE (CMC‐NE‐CEO) of CEO at selected concentration of CEO 0.9708%. CEO, cinnamon essential oil; CMC, carboxymethyl cellulose; ME, Macro‐emulsion; NE, Nano‐emulsion

Microstructure analysis was carried out for better understanding the effects of different formulations on structural, mechanical and barrier‐related properties of the emulsified films. The surface and the cross‐section microstructure of the emulsified films are shown in Figures 1 and 2, respectively.

Both pure CMC‐based films and the films loaded with the CEO Nano‐emulsions displayed a continuous, smooth and flat surface. On the contrary, the films containing Macro‐emulsions showed a more heterogeneous structure and an irregularity on a rough surface. These differences in the surface structures could be related to the different dimensions of the essential oil droplets in the film matrix due to different rates of emulsion destabilizing phenomena such as flocculation, coalescence and creaming. The Nano‐scale droplets in comparison with the Macro ones, showed better stability against these phenomena due to higher zeta potential, smaller droplet size and narrower size distribution. Therefore, the interactions between the biopolymer matrix and the CEO Nano‐droplets could be increased, inhibiting phase separation during the drying step (Acevedo‐Fani et al., 2015; Fabra et al., 2011; Vargas et al., 2009).

The *SEM* images from the cross‐section of the Nano‐emulsion‐loaded films revealed uniform, dense and compact layers (Figure 2). However, a porous texture with sponge‐like structure was observed in the Macro‐emulsion‐loaded films. This might be attributed to the droplet flocculation and coalescence, leading to a larger droplet size and size distribution. Macro‐droplets could leach upwards toward the film surface during the drying due to creaming and Micro‐pores, cavities and cracks forming throughout the matrix. This unsteady texture with a lot of pores and holes could negatively affect the mechanical and barrier properties of the resulted films (Villalobos et al., 2005).

### 3.4 Atomic force microscopy (AFM)

**Figure 3:**
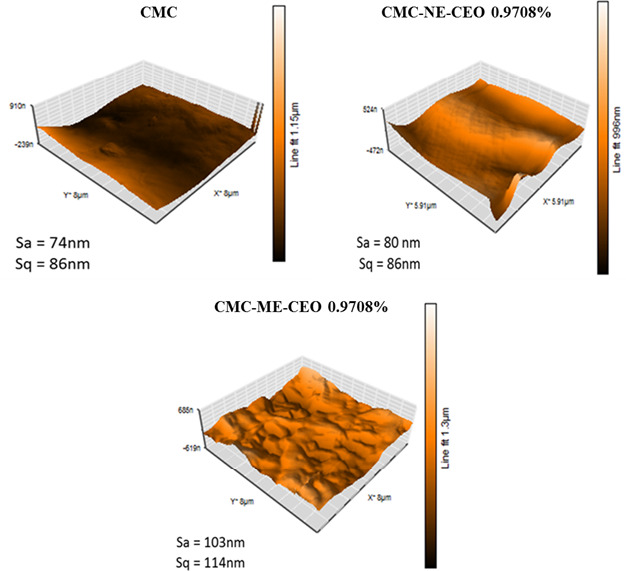
Atomic force microscopy (AFM) images of the surface topography of the CMC control film and films loaded with ME (CMC‐ME‐CEO) and NE (CMC‐NE‐CEO) of CEO at selected concentration of CEO 0.9708%. CEO, cinnamon essential oil; CMC, carboxymethyl cellulose; ME, Macro‐emulsion; NE, Nano‐emulsion

The roughness of the films is often critical to their applications, and in particular, roughness strongly impacts their optical, barrier and frictional properties. According to *SEM* surface micrographs, both control and Nano‐emulsion‐loaded films were smoother than Macro‐emulsion‐loaded ones. AFM 3D‐plot images were used for further investigation on the surface morphological differences between the Macro and Nano‐emulsion‐incorporated films during the drying. According to Figure 3, the CEO‐free film showed smoother surface and Sa and Sq roughness parameters were 74 and 86 nm, respectively. Approximately similar values were obtained for the NE‐loaded films. However, Sa and Sq values in the ME‐loaded films increased significantly to 103 and 114 nm, respectively.

These results accentuated that embedding the CEO inside the CMC matrix was improved by Nano‐emulsification technique. These results are in agreement with the findings of Acevedo‐Fani et al. (2015) regarding alginate‐based edible films incorporated with thyme, lemongrass and sage essential oils. They reported that Nano‐droplets showed relatively regular surfaces in comparison with the droplets in the emulsions with larger droplet size due to the homogeneous entrapment in the matrix and lower migration of oil droplets toward the film surface. Furthermore, similar structure was reported by Atarés et al. (2010) for sodium caseinate‐based edible film incorporated with cinnamon essential oil.

### 3.5 Thickness of the films

Thickness values of the CMC‐based films incorporated with the Macro and Nano‐emulsions of CEO at various concentrations are given in Table 2. When Macro‐emulsion was incorporated in the film matrix, the thickness of the films increased nonsignificantly (*p* > .05) from 0.07 mm in the control film to 0.080, 0.106 and 0.093 mm at the CEO concentrations of 0.2427%, 0.4854% and 0.9708%, respectively. However, the addition of CEO as a Nano‐emulsion with concentrations of 0.4854% and 0.9708% increased the thickness of the films significantly (*p* < .05) to 0.113 and 0.126 mm, respectively. Han and Krochta (1999) reported that film thickness was influenced by the solid content of the film‐forming solution. In the film‐forming solutions, probably due to extensive flocculation of Macro‐droplets during the drying step and subsequently the creaming destabilization, CEO could be leached out of the surface in dried films. This destabilization phenomenon was intensified by increasing the CEO content in the ME‐loaded film; hence, the thickness value of the film at high concentration (CEO 0.9708%) was lower than the intermediate concentration (CEO 0.4854%).

**Table 2:** Thickness, Ultimate Tensile strength (UTS) and Strain at break point (SAB) of control and CMC films incorporated with Macro and Nano‐emulsions of CEO at different concentrations

Film types	Thickness (mm)	UTS (MPa)	SAB (%)
CMC	0.070 ± 0.005^c^	16.41 ± 0.01^a^	53.56 ± 1.08^d^
CMC‐ME‐CEO 0.2427%	0.080 ± 0.017^c^	8.96 ± 3.04^b^	55.60 ± 0.34^d^
CMC‐ME‐CEO 0.4854%	0.106 ± 0.025^abc^	7.60 ± 3.02^b^	67.12 ± 1.23^c^
CMC‐ME‐CEO 0.9708%	0.093 ± 0.005^bc^	5.84 ± 2.78^b^	80.0 ± 1.06^b^
CMC‐NE‐CEO 0.2427%	0.08 ± 0.026^c^	6.46 ± 0.69^b^	58.62 ± 3.76^cd^
CMC‐NE‐CEO 0.4854%	0.113 ± 0.005^ab^	6.25 ± 0.05^b^	82.05 ± 1.76^b^
CMC‐NE‐CEO 0.9708%	0.126 ± 0.011^a^	6.05 ± 2.39^b^	94.77 ± 2.35^a^

Note

Values were given as mean ± standard deviations. Different superscripts in the same column indicate significant differences (*p* < .05).

In the NE‐loaded films, the CEO droplets likely remained stable in the matrix and did not suffer from extensive destabilization phenomena during the drying which in turn increased the solid content in the final film; therefore, they were significantly thicker than the ME‐loaded and control films. These results are in agreement with the observations of Perdones et al. (2014) who noted that the thickness of the chitosan films incorporated with the cinnamon leaf oil‐oleic acid was higher than those of the films with cinnamon leaf oil only. They described that oleic acid had an encapsulating effect on the oil droplet, reducing losses of the oil droplets during the drying step.

### 3.6 Mechanical properties

Ultimate tensile strength (resistance to elongation) and strain at break point (capacity for stretching) are two important mechanical properties of the films obtained from true stress (*σ*) versus Hencky (*ɛ*_h_) strain curves (McHugh & Krochta, 1994).

**Figure 4:**
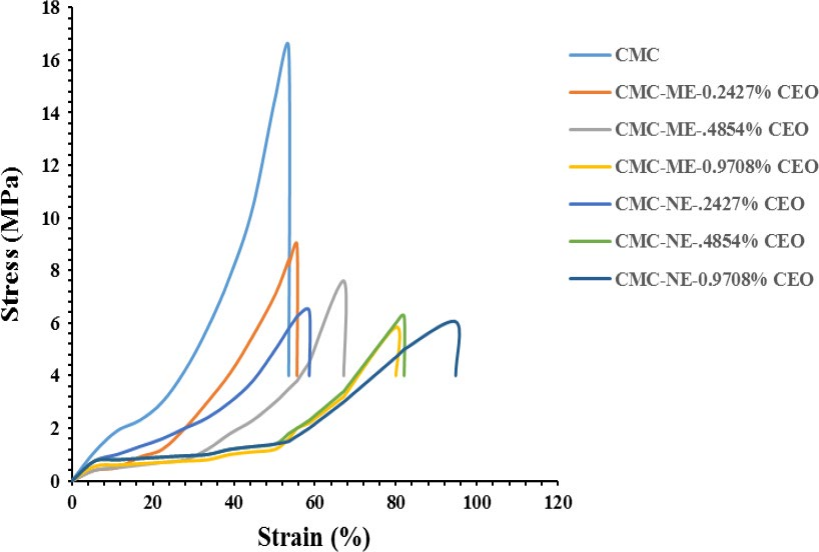
Representative stress–strain curves of the films

Regardless of the type and the droplet size of the emulsion, the incorporation of CEO had a plasticizing effect on the CMC‐based films. Figure 4 shows representative stress–strain curves of the films. According to Table 2, the emulsified films had significantly lower UTS and higher SAB in comparison with the control film (*p* < .05). Incorporating essential oils probably weakened the network structure via interruption and substitution of stronger biopolymer intermolecular interactions with weaker biopolymer essential oils. Regardless of the mechanism, the emulsified films presented lower UTS values than the high‐density polyethylene (HDPE; typically 22–23 MPa) and the low‐density polyethylene (LDPE; typically 19–44 MPa) materials which are commonly used in commercial packaging. The UTS values between the Macro and Nano‐emulsion‐loaded films were not significantly different (*p* > .05) in all the concentrations of the CEO. However, there was a significant difference (*p* < .05) between SAB values of the ME and NE‐loaded films in the CEO concentrations of 0.4854% and 0.9708% (67.12% and 80% in the ME film vs. 82.05% and 94.77% in the NE films). These results showed that the reduction of the oil droplets size to Nano‐metric scale caused more extensibility and flexibility in the NE films in comparison with the ME ones. This phenomenon could be attributed to the further interruption of the biopolymer–biopolymer interactions due to higher specific surface area of Nano‐droplets (Acevedo‐Fani et al., 2015; Otoni, Moura, et al., 2014; Otoni, Pontes, et al., 2014), and lower leaching out of the CEO onto the film surface in the NE‐loaded films (Acevedo‐Fani et al., 2015). The current results for SAB were in agreement with previous researches, showing that the reduction in size of *Zataria multiflora* EO droplets increased the interaction between EO Nano‐droplets and basil seed gum, resulting in an enhanced stretching ability in the emulsified films (Hashemi Gahruie et al., 2017). Nevertheless, the SAB of the films prepared here was substantially lower than those of the HDPE (150%) and the LDPE (400%) (Crompton, 2012).

### 3.7 Water vapor permeability (WVP)

**Figure 5:**
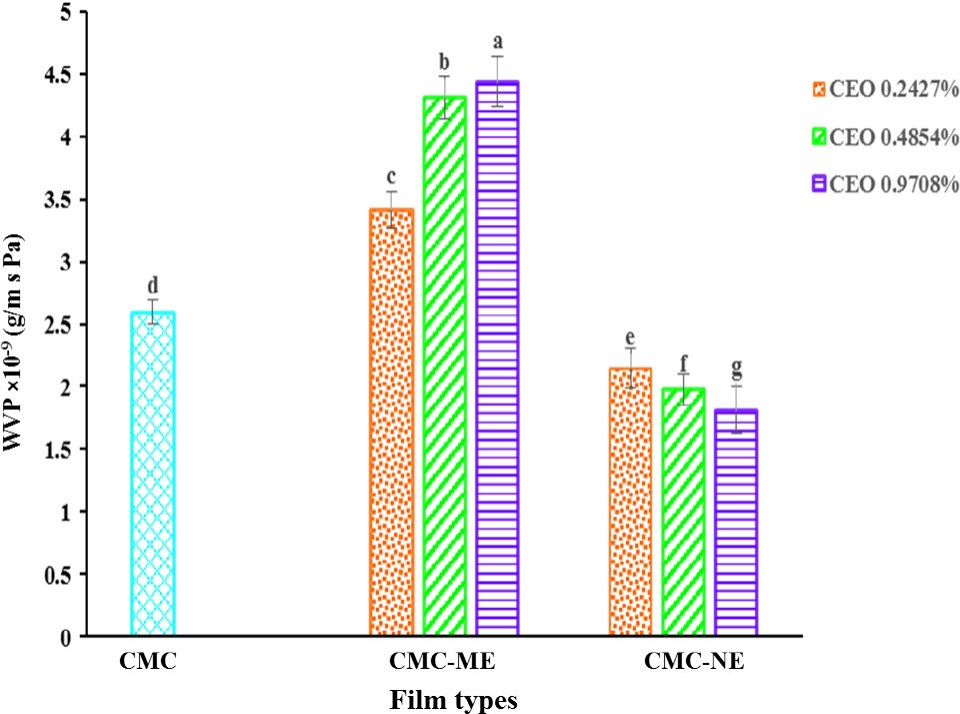
Water vapor permeability (WVP) of the control CMC film and films loaded with ME and NE of CEO at different concentrations. Bars with different letters represent statistical differences (*p* < .05). CEO, cinnamon essential oil; CMC, carboxymethyl cellulose; ME, Macro‐emulsion; NE, Nano‐emulsion

In order to prevent the loss of quality in food products during storage, packaging films should have suitable barrier properties against moisture transmission from food to environment or vice versa. Hence, WVP is an important factor in the determination of the functional properties of the edible films (Ma et al., 2008). The effects of Macro and Nano‐emulsion incorporation on the WVP of the CMC‐based films are presented in Figure 5. The WVP of the control film was 2.59 × 10^–9^ g/ms Pa and the addition of the CEO ME caused a significant increase (*p* < .05) to 3.4 × 10^–9^ and 4.43 × 10^–9^ g/ms Pa for the CEO concentrations of 0.2427% and 0.9708%, respectively. However, the films containing the CEO NE showed significantly (*p* < .05) lower WVP in comparison with the control film (2.14 × 10^–9^ and 1.80 × 10^–9^ g/ms Pa for the CEO concentrations of 0.2427% and 0.9708%, respectively). This indicated that the reduction of the CEO droplet size from Macro to Nano size had an important role in reducing water vapor transmission throughout the biopolymer matrix. The WVP values obtained here were higher than those obtained for the cellophane (8.4 × 10^–11^ g/ms Pa), the HDPE (2.31 × 10^–13^ g/ms Pa), and the LDPE (9.14 × 10^–13^ g/ms Pa) (Hosseini et al., 2015). The WVP of the films depends on both solubility and diffusibility of the water molecules in the film matrix. The essential oils are nonpolar compounds which could reduce the solubility factor by increasing the hydrophobic nature of the polymer matrix; however, they could increase or decrease the diffusibility parameter which depends on the lipid type, the compatibility and the distribution of the oil droplets in the polymer matrix. Essential oil has plasticizing effects, possibly making the matrix more open, increasing the diffusibility of water molecules. On the other hand, the formation of an interconnecting lipid network within the film matrix creates a tortuous pathway through the film for the water molecules. Therefore, the final effects of the essential oil on WVP depend on the dominant mechanism (Han et al., 2018; Rodrigues et al., 2018).

The lower WVP in the films containing the CEO NE, could be due to the existence of a compact and dense structure (according to *SEM* micrograph), a higher thickness and a homogeneous dispersion of the oil droplets, resulting in a uniform hydrophobicity in the matrix.

The high droplet size and polydispersity index of ME could increase the destabilization phenomena such as flocculation and creaming during the drying process, likely leading to the formation of Micro‐pores throughout the films and hence, greater diffusion of the water molecules. In addition, phase separation could decrease hydrophobicity in some parts of the polymer matrix. This destabilization phenomenon was accelerated in the high CEO concentrations. Similar results were reported by Dashipour et al. (2015) who observed that increasing the concentration of *Zataria multiflora* essential oil improved the WVP of the CMC‐based films. They suggested that the aggregation of the Micro‐droplets caused the reduction of the structure cohesiveness which in turn facilitated water molecule diffusion throughout the films.

Thus, the reduction and the homogeneous distribution of the particle size or the oil droplets could significantly reduce the water vapor permeability of the emulsion‐based films. Also these results were in agreement with the data obtained from studies on isolated soy protein/carvacrol essential oil and cinnamaldehyde (Otoni et al., 2016), alginate/sage, thyme and lemongrass essential oils (Acevedo‐Fani et al., 2015) and chitosan/oleic acid (Vargas et al., 2011).

### 3.8 Antifungal properties

When natural preservatives such as plant essential oils are used directly in the form of emulsion in food products, they usually show unfavorable effects on sensorial properties. Therefore, their entrapment in the edible film matrix or their encapsulation could lead to the less usage of the raw material and the better maintenance of the antimicrobial efficiency, hence prolonging the shelf life of different food products.

The antifungal activities of the cinnamon essential oil (CEO) are related to major phenolic or aldehyde components such as eugenol and cinnamaldehyde. Eugenol destroys microbial cells likely by two important mechanisms; the leaching out the cell content by increasing the permeability of the cellular membranes and releasing protons of the hydroxyl groups, resulting in the reduction of the proton gradient and in turn the depletion of the energy pools in the microbial cells (Ben Afra et al., 2006). Another effective mechanism of cinnamaldehyde in preventing microbial growth is related to the nucleophilic compound in the microorganisms' structure and the highly electrophilic compounds such as the carbonyl groups in the cinnamaldehyde structure (Neri et al., 2006).

The antifungal activity of the CMC‐based films containing ME or NE of the CEO at different concentrations was evaluated against *A. niger* and *M. racemous,* using disk inhibition test. The data are summarized in Table 3. As expected, the CEO‐free films were not able to inhibit the fungal growth in the film disk area. With an increase in the CEO concentration, the antifungal index increased in both the ME and the NE‐loaded films. The antifungal index of the ME films increased from 14.16% and 20.82% to 18.81% and 25% for *A. niger* and *M. racemous* at the CEO concentration of 0.9708%, respectively. The reduction of the CEO droplet size from ME to NE‐scale had an important effect in improving antifungal properties against *A.niger* or *M.racemous* and greater inhibition haloes could be observed in the NE‐loaded films in comparison with the ME‐loaded ones. This difference was more significant in higher concentrations of the CEO in the films. Several mechanisms have been reported for the higher antifungal effect of the NE‐loaded films in comparison with the ME ones, such as (a) reducing the selective permeability of the cellular membranes against mass transfer due to an increased passive absorption mechanism in microbial cells (Chen et al., 2016), (b) increasing both the concentrations and the bioavailability of the bioactive compounds in plasma by facilitating the delivery of Nano‐active compounds through cell membranes (Huang et al., 2010). In another research on the effect of the Nano‐emulsion of *Zataria multiflora* essential oil on the growth of selected bacteria, it was shown that the reduction of the droplet size by Ultrasonication led to a better growth inhibition (Hashemi Gahruie et al., 2017).

**Table 3:** Inhibition diameter and Antifungal Index percentage (A.I) of *A. niger* and *M. racemous* in control and films containing ME or NE of CEO at different concentrations after 24 hr at 25°C

Film types	Inhibition diameter of *A. niger* (mm)	Inhibition diameter of *M. racemous* (mm)	A.I of *A. niger* (%)	A.I of *M. racemous* (%)
CMC	0^d^	0^c^	0^d^	0^c^
CMC‐ME‐CEO 0.2427%	9.5 ± 0.01^c^	0^c^	11.87^c^	0^c^
CMC‐ME‐CEO 0.4854%	10.38 ± 0.09^c^	13.33 ± 0.77^b^	12.97^c^	16.66^b^
CMC‐ME‐CEO 0.9708%	11.33 ± 0.01^bc^	16.66 ± 2.88^ab^	14.16^bc^	20.82^ab^
CMC‐NE‐CEO 0.2427%	11.5 ± 0.01^bc^	15 ± 0.01^ab^	14.37^bc^	18.75^ab^
CMC‐NE‐CEO 0.4854%	13.16 ± 1.89^b^	16.66 ± 2.88^ab^	16.45^b^	20.82^ab^
CMC‐NE‐CEO 0.9708%	15.05 ± 2.08^a^	20 ± 0.01^a^	18.81^a^	25^a^

Note

Values were given as mean ± standard deviations. Different superscripts in the same column indicate significant differences (*p* < .05).

## 4 CONCLUSION

Comparing the different physicochemical and antifungal properties of the CMC‐based active films containing Macro and Nano‐emulsions of CEO showed significant differences. The reduction of the CEO emulsion droplet size to few tens of nanometers by sonication improved the antifungal efficiency of the emulsified films. According to the microstructural analyses, Nano‐emulsion‐based films had a homogenous morphology and no phase separation was observed. The reduction of the oil droplet size increased the flexibility of the films significantly. Also, the water barrier properties of the emulsion films were negatively affected by the oil droplet size. This study could lead to the development of new Nano‐active emulsified films by combining Nano‐emulsion and active packaging concepts.

## Data Availability

Data available on request from the authors.
